# Poly[(μ_4_-benzene-1,3,5-tricarboxyl­ato)bis­(*N*,*N*-dimethyl­formamide)­cerium(III)]

**DOI:** 10.1107/S160053681102277X

**Published:** 2011-07-06

**Authors:** Zhongyue Li, Kun Liu

**Affiliations:** a The Department of Physics–Chemistry, Henan Polytechnic University, Jiaozuo 454000, People’s Republic of China

## Abstract

The asymmetric unit of the title rare earth coordination polymer, [Ce(C_9_H_3_O_6_)(C_3_H_7_NO)_2_]_*n*_, contains one eight-coordinated Ce^3+^ ion, one benzene-1,3,5-tricarboxyl­ate (BTC) ligand and two coordinated *N*,*N*-dimethyl­formamide (DMF) mol­ecules. The Ce^3+^ ion is coordinated by six O atoms from four carboxyl­ate groups of the BTC ligands and by two O atoms from two terminal DMF mol­ecules.

## Related literature

Metal-organic framework (MOF) design and construction is currently a flourishing field of research owing to the intriguing mol­ecular topologies and the potentially exploitable adsorption, catalytic, fluorescence, and magnetic properties, see: Chen *et al.* (2006 ▶); Serre *et al.* (2007 ▶); Zhang *et al.* (2007 ▶). As functional metal centers, rare earth metals are attracting increasing attention from synthesis chemists for their coordination properties and special chemical characteristics arising from 4f electrons and their propensity to form isostructural complexes, see: Thirumurugan *et al.* (2004 ▶); Long *et al.* (2001 ▶).
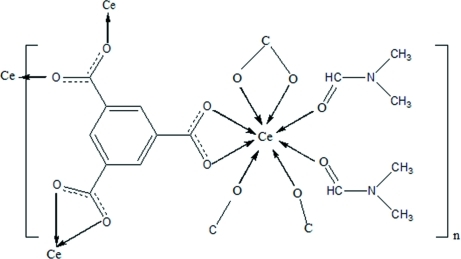


## Experimental

### 

#### Crystal data

[Ce(C_9_H_3_O_6_)(C_3_H_7_NO)_2_]
*M* *_r_* = 493.43
Monoclinic, 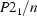

*a* = 10.6994 (11) Å
*b* = 13.6773 (14) Å
*c* = 12.1961 (13) Å
β = 101.574 (2)°
*V* = 1748.5 (3) Å^3^
*Z* = 4
Mo *K*α radiation
μ = 2.65 mm^−1^
*T* = 298 K
0.8 × 0.6 × 0.5 mm

#### Data collection

Bruker SMART CCD area-detector diffractometer
Absorption correction: multi-scan (*SADABS*; Bruker, 2001 ▶) *T*_min_ = 0.226, *T*_max_ = 0.351
9223 measured reflections
3087 independent reflections
2516 reflections with *I* > 2σ(*I*)
*R*_int_ = 0.059

#### Refinement

*R*[*F* ^2^ > 2σ(*F* ^2^)] = 0.046
*wR*(*F* ^2^) = 0.076
*S* = 1.02
3087 reflections
239 parameters
H-atom parameters constrained
Δρ_max_ = 1.14 e Å^−3^
Δρ_min_ = −1.05 e Å^−3^

### 

Data collection: *SMART* (Bruker, 2001 ▶); cell refinement: *SAINT* (Bruker, 2001 ▶); data reduction: *SAINT*; program(s) used to solve structure: *SHELXS97* (Sheldrick, 2008 ▶); program(s) used to refine structure: *SHELXL97* (Sheldrick, 2008 ▶); molecular graphics: *SHELXTL* (Sheldrick, 2008 ▶); software used to prepare material for publication: *SHELXL97*.

## Supplementary Material

Crystal structure: contains datablock(s) I, global. DOI: 10.1107/S160053681102277X/qm2010sup1.cif

Structure factors: contains datablock(s) I. DOI: 10.1107/S160053681102277X/qm2010Isup2.hkl

Additional supplementary materials:  crystallographic information; 3D view; checkCIF report

## Figures and Tables

**Fig. 1.**
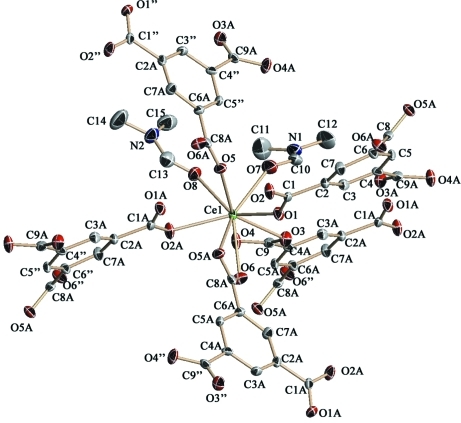
Coordination environment of Ce in the compound with nonhydrogen atoms represented by thermal ellipsoids draw at 30 % probability level.

## supplementary crystallographic information

### Experimental

All reagents were of analytical grade. A mixture of cerium nitrate (40 mg, 0.10 mmol) and *µ_3_*-benzene-*1,3,5*-tricarboxylate acid(H_3_BTC) (10 mg, 0.05 mmol) was dissolved in *N,N'*-dimethylformamide (DMF) (10 mL) and isopropanol (2 mL) at room temperature, two drops of triethylamine was added, then some nitric acid(2M) was added utill the solution is clear. This mixture was placed in a 20 mL test tube. Then a small vial containing triethylamine (0.1 mL) and DMF (1.5 mL) was put in the test tube. The test tube was left undisturbed at room temprature for 15 days.

### Refinement

H atoms were positioned geometrically, with C—H = 0.93 Å.

### Crystal data

**Table:**

[Ce(C_9_H_3_O_6_)(C_3_H_7_NO)_2_]	*Z* = 4
*M**_r_* = 493.43	*F*(000) = 972
Monoclinic, *P*2_1_/*n*	*D*_x_ = 1.874 Mg m^−^^3^
*a* = 10.6994 (11) Å	Mo *K*α radiation, λ = 0.71073 Å
*b* = 13.6773 (14) Å	µ = 2.65 mm^−^^1^
*c* = 12.1961 (13) Å	*T* = 298 K
β = 101.574 (2)°	Rod, colorless
*V* = 1748.5 (3) Å^3^	0.8 × 0.6 × 0.5 mm

### Data collection

**Table:**

Bruker SMART CCD area-detector diffractometer	3087 independent reflections
Radiation source: fine-focus sealed tube	2516 reflections with *I* > 2σ(*I*)
graphite	*R*_int_ = 0.059
φ and ω scans	θ_max_ = 25.0°, θ_min_ = 2.3°
Absorption correction: multi-scan (*SADABS*; Bruker, 2001)	*h* = −9→12
*T*_min_ = 0.226, *T*_max_ = 0.351	*k* = −16→16
9223 measured reflections	*l* = −13→14

### Refinement

**Table:**

Refinement on *F*^2^	Primary atom site location: structure-invariant direct methods
Least-squares matrix: full	Secondary atom site location: difference Fourier map
*R*[*F*^2^ > 2σ(*F*^2^)] = 0.046	Hydrogen site location: inferred from neighbouring sites
*wR*(*F*^2^) = 0.076	H-atom parameters constrained
*S* = 1.01	*w* = 1/[σ^2^(*F*_o_^2^) + (0.0173*P*)^2^] where *P* = (*F*_o_^2^ + 2*F*_c_^2^)/3
3087 reflections	(Δ/σ)_max_ = 0.008
239 parameters	Δρ_max_ = 1.14 e Å^−^^3^
0 restraints	Δρ_min_ = −1.05 e Å^−^^3^

### Special details

**Table:**

Geometry. All e.s.d.'s (except the e.s.d. in the dihedral angle between two l.s. planes) are estimated using the full covariance matrix. The cell e.s.d.'s are taken into account individually in the estimation of e.s.d.'s in distances, angles and torsion angles; correlations between e.s.d.'s in cell parameters are only used when they are defined by crystal symmetry. An approximate (isotropic) treatment of cell e.s.d.'s is used for estimating e.s.d.'s involving l.s. planes.
Refinement. Refinement of *F*^2^ against ALL reflections. The weighted *R*-factor *wR* and goodness of fit *S* are based on *F*^2^, conventional *R*-factors *R* are based on *F*, with *F* set to zero for negative *F*^2^. The threshold expression of *F*^2^ > σ(*F*^2^) is used only for calculating *R*-factors(gt) *etc*. and is not relevant to the choice of reflections for refinement. *R*-factors based on *F*^2^ are statistically about twice as large as those based on *F*, and *R*- factors based on ALL data will be even larger.

### Fractional atomic coordinates and isotropic or equivalent isotropic displacement parameters (Å^2^)

**Table:**

	*x*	*y*	*z*	*U*_iso_*/*U*_eq_	
Ce1	0.62306 (3)	1.03609 (3)	0.39566 (3)	0.01481 (11)	
C1	0.6993 (6)	0.9240 (4)	0.6584 (5)	0.0170 (15)	
C2	0.8218 (6)	0.8724 (4)	0.7133 (6)	0.0188 (15)	
C3	0.9313 (6)	0.8882 (4)	0.6695 (5)	0.0189 (15)	
H3	0.9285	0.9309	0.6096	0.023*	
C4	1.0448 (6)	0.8403 (4)	0.7153 (5)	0.0174 (15)	
C5	1.0469 (6)	0.7730 (4)	0.7994 (5)	0.0179 (15)	
H5	1.1210	0.7378	0.8264	0.021*	
C6	0.9388 (6)	0.7571 (4)	0.8446 (5)	0.0178 (15)	
C7	0.8280 (6)	0.8097 (4)	0.8014 (5)	0.0206 (15)	
H7	0.7567	0.8018	0.8334	0.025*	
C8	0.9393 (6)	0.6846 (5)	0.9368 (6)	0.0184 (15)	
C9	0.8386 (6)	1.1342 (4)	0.3310 (6)	0.0189 (15)	
C10	0.8855 (7)	0.8868 (5)	0.3589 (7)	0.0347 (19)	
H10	0.9423	0.8752	0.4260	0.042*	
C11	0.8492 (8)	0.9013 (7)	0.1589 (7)	0.068 (3)	
H11A	0.7749	0.9368	0.1690	0.102*	
H11B	0.8236	0.8408	0.1212	0.102*	
H11C	0.8959	0.9396	0.1148	0.102*	
C12	1.0614 (7)	0.8523 (6)	0.2683 (8)	0.061 (3)	
H12A	1.1029	0.9018	0.2327	0.091*	
H12B	1.0622	0.7915	0.2290	0.091*	
H12C	1.1057	0.8444	0.3445	0.091*	
C13	0.4387 (8)	0.9287 (5)	0.1597 (7)	0.038 (2)	
H13	0.3729	0.9378	0.1982	0.046*	
C14	0.2840 (8)	0.8444 (6)	0.0187 (8)	0.068 (3)	
H14A	0.2850	0.7743	0.0206	0.102*	
H14B	0.2575	0.8662	−0.0573	0.102*	
H14C	0.2255	0.8684	0.0627	0.102*	
C15	0.5082 (8)	0.8594 (6)	0.0012 (7)	0.053 (2)	
H15A	0.5838	0.8965	0.0303	0.080*	
H15B	0.4775	0.8761	−0.0759	0.080*	
H15C	0.5278	0.7909	0.0072	0.080*	
N1	0.9291 (6)	0.8814 (4)	0.2665 (6)	0.0356 (16)	
N2	0.4117 (6)	0.8815 (4)	0.0643 (5)	0.0374 (16)	
O1	0.7104 (4)	0.9890 (3)	0.5883 (4)	0.0250 (11)	
O2	0.5978 (4)	0.9001 (3)	0.6867 (4)	0.0263 (11)	
O3	0.8522 (4)	1.1009 (3)	0.4277 (4)	0.0268 (11)	
O4	0.7289 (4)	1.1428 (3)	0.2671 (4)	0.0289 (12)	
O5	0.5116 (4)	0.8900 (3)	0.4415 (4)	0.0241 (11)	
O6	0.6335 (4)	1.1973 (3)	0.4976 (4)	0.0270 (12)	
O7	0.7738 (5)	0.9063 (3)	0.3633 (4)	0.0406 (14)	
O8	0.5433 (4)	0.9618 (4)	0.2023 (4)	0.0342 (12)	

### Atomic displacement parameters (Å^2^)

**Table:**

	*U*^11^	*U*^22^	*U*^33^	*U*^12^	*U*^13^	*U*^23^
Ce1	0.01096 (19)	0.01648 (19)	0.0170 (2)	−0.00043 (18)	0.00285 (13)	−0.00011 (19)
C1	0.012 (4)	0.018 (4)	0.019 (4)	0.001 (3)	−0.001 (3)	−0.005 (3)
C2	0.012 (4)	0.017 (4)	0.026 (4)	0.001 (3)	0.000 (3)	−0.002 (3)
C3	0.020 (4)	0.016 (3)	0.021 (4)	−0.001 (3)	0.004 (3)	0.006 (3)
C4	0.020 (4)	0.011 (3)	0.021 (4)	−0.001 (3)	0.005 (3)	0.002 (3)
C5	0.010 (4)	0.017 (4)	0.025 (4)	0.000 (3)	−0.001 (3)	0.004 (3)
C6	0.016 (4)	0.015 (4)	0.022 (4)	0.002 (3)	0.003 (3)	0.003 (3)
C7	0.016 (4)	0.026 (4)	0.021 (4)	0.001 (3)	0.005 (3)	0.005 (3)
C8	0.013 (4)	0.016 (4)	0.026 (4)	−0.005 (3)	0.003 (3)	−0.002 (3)
C9	0.015 (4)	0.015 (4)	0.029 (4)	−0.004 (3)	0.009 (3)	0.000 (3)
C10	0.032 (5)	0.024 (4)	0.047 (6)	0.007 (4)	0.004 (4)	−0.011 (4)
C11	0.068 (7)	0.096 (8)	0.037 (6)	0.023 (6)	0.006 (5)	−0.025 (6)
C12	0.032 (5)	0.060 (6)	0.097 (8)	0.011 (4)	0.029 (5)	−0.020 (6)
C13	0.044 (5)	0.041 (5)	0.029 (5)	0.003 (4)	0.004 (4)	0.001 (4)
C14	0.069 (7)	0.071 (7)	0.053 (7)	−0.018 (5)	−0.014 (5)	−0.015 (5)
C15	0.075 (7)	0.043 (5)	0.039 (6)	−0.004 (4)	0.006 (5)	−0.006 (4)
N1	0.029 (4)	0.030 (4)	0.049 (5)	0.005 (3)	0.011 (3)	−0.013 (3)
N2	0.043 (4)	0.040 (4)	0.024 (4)	−0.005 (3)	−0.004 (3)	−0.005 (3)
O1	0.023 (3)	0.024 (3)	0.025 (3)	0.002 (2)	−0.002 (2)	0.010 (2)
O2	0.012 (3)	0.039 (3)	0.029 (3)	0.003 (2)	0.005 (2)	0.008 (2)
O3	0.021 (3)	0.036 (3)	0.026 (3)	−0.002 (2)	0.008 (2)	0.004 (2)
O4	0.012 (3)	0.042 (3)	0.032 (3)	0.003 (2)	0.003 (2)	0.009 (2)
O5	0.019 (3)	0.019 (3)	0.035 (3)	−0.004 (2)	0.007 (2)	−0.007 (2)
O6	0.030 (3)	0.025 (3)	0.030 (3)	−0.008 (2)	0.016 (2)	−0.007 (2)
O7	0.028 (3)	0.036 (3)	0.060 (4)	0.003 (2)	0.015 (3)	−0.012 (3)
O8	0.031 (3)	0.036 (3)	0.031 (3)	0.002 (3)	−0.002 (2)	−0.011 (3)

### Geometric parameters (Å, °)

**Table:**

Ce1—O1	2.434 (4)	C9—O3	1.245 (8)
Ce1—O5	2.448 (4)	C9—O4	1.278 (7)
Ce1—O7	2.483 (5)	C9—C4^iii^	1.511 (8)
Ce1—O6	2.522 (4)	C10—O7	1.236 (7)
Ce1—O2^i^	2.531 (4)	C10—N1	1.306 (9)
Ce1—O8	2.553 (5)	C10—H10	0.9300
Ce1—O3	2.562 (4)	C11—N1	1.441 (10)
Ce1—O4	2.565 (4)	C11—H11A	0.9600
Ce1—O5^i^	2.862 (4)	C11—H11B	0.9600
Ce1—C9	2.910 (6)	C11—H11C	0.9600
Ce1—C8^ii^	3.049 (6)	C12—N1	1.466 (8)
Ce1—Ce1^i^	4.1322 (7)	C12—H12A	0.9600
C1—O2	1.247 (7)	C12—H12B	0.9600
C1—O1	1.256 (7)	C12—H12C	0.9600
C1—C2	1.520 (8)	C13—O8	1.222 (8)
C2—C7	1.367 (8)	C13—N2	1.311 (9)
C2—C3	1.399 (8)	C13—H13	0.9300
C3—C4	1.394 (8)	C14—N2	1.459 (9)
C3—H3	0.9300	C14—H14A	0.9600
C4—C5	1.374 (8)	C14—H14B	0.9600
C4—C9^iii^	1.511 (8)	C14—H14C	0.9600
C5—C6	1.394 (8)	C15—N2	1.438 (9)
C5—H5	0.9300	C15—H15A	0.9600
C6—C7	1.397 (8)	C15—H15B	0.9600
C6—C8	1.498 (8)	C15—H15C	0.9600
C7—H7	0.9300	O2—Ce1^i^	2.531 (4)
C8—O6^iv^	1.236 (7)	O5—C8^vi^	1.276 (7)
C8—O5^v^	1.276 (7)	O5—Ce1^i^	2.862 (4)
C8—Ce1^iv^	3.049 (6)	O6—C8^ii^	1.236 (7)
			
O1—Ce1—O5	70.96 (14)	C4—C3—H3	119.9
O1—Ce1—O7	80.11 (16)	C2—C3—H3	119.9
O5—Ce1—O7	79.29 (15)	C5—C4—C3	119.8 (6)
O1—Ce1—O6	77.59 (14)	C5—C4—C9^iii^	122.9 (6)
O5—Ce1—O6	125.18 (13)	C3—C4—C9^iii^	117.3 (6)
O7—Ce1—O6	137.51 (16)	C4—C5—C6	120.6 (6)
O1—Ce1—O2^i^	128.43 (14)	C4—C5—H5	119.7
O5—Ce1—O2^i^	85.03 (14)	C6—C5—H5	119.7
O7—Ce1—O2^i^	140.18 (16)	C5—C6—C7	118.7 (6)
O6—Ce1—O2^i^	80.79 (15)	C5—C6—C8	121.4 (5)
O1—Ce1—O8	141.15 (15)	C7—C6—C8	119.9 (5)
O5—Ce1—O8	78.37 (15)	C2—C7—C6	121.5 (6)
O7—Ce1—O8	71.08 (16)	C2—C7—H7	119.2
O6—Ce1—O8	140.98 (15)	C6—C7—H7	119.2
O2^i^—Ce1—O8	70.00 (14)	O6^iv^—C8—O5^v^	122.6 (6)
O1—Ce1—O3	76.95 (14)	O6^iv^—C8—C6	119.1 (6)
O5—Ce1—O3	137.95 (14)	O5^v^—C8—C6	118.3 (5)
O7—Ce1—O3	68.89 (15)	O6^iv^—C8—Ce1^iv^	53.7 (3)
O6—Ce1—O3	71.07 (14)	O5^v^—C8—Ce1^iv^	69.4 (3)
O2^i^—Ce1—O3	136.88 (14)	C6—C8—Ce1^iv^	167.4 (4)
O8—Ce1—O3	114.27 (14)	O3—C9—O4	122.0 (6)
O1—Ce1—O4	127.54 (14)	O3—C9—C4^iii^	119.4 (6)
O5—Ce1—O4	153.98 (15)	O4—C9—C4^iii^	118.5 (6)
O7—Ce1—O4	85.95 (15)	O3—C9—Ce1	61.5 (3)
O6—Ce1—O4	79.79 (14)	O4—C9—Ce1	61.7 (3)
O2^i^—Ce1—O4	93.02 (14)	C4^iii^—C9—Ce1	165.2 (4)
O8—Ce1—O4	76.61 (15)	O7—C10—N1	124.5 (8)
O3—Ce1—O4	50.97 (14)	O7—C10—H10	117.8
O1—Ce1—O5^i^	64.72 (13)	N1—C10—H10	117.8
O5—Ce1—O5^i^	78.09 (14)	N1—C11—H11A	109.5
O7—Ce1—O5^i^	142.78 (15)	N1—C11—H11B	109.5
O6—Ce1—O5^i^	47.78 (12)	H11A—C11—H11B	109.5
O2^i^—Ce1—O5^i^	66.03 (13)	N1—C11—H11C	109.5
O8—Ce1—O5^i^	131.33 (13)	H11A—C11—H11C	109.5
O3—Ce1—O5^i^	111.80 (13)	H11B—C11—H11C	109.5
O4—Ce1—O5^i^	124.65 (13)	N1—C12—H12A	109.5
O1—Ce1—C9	102.23 (17)	N1—C12—H12B	109.5
O5—Ce1—C9	152.48 (15)	H12A—C12—H12B	109.5
O7—Ce1—C9	73.23 (16)	N1—C12—H12C	109.5
O6—Ce1—C9	76.78 (15)	H12A—C12—H12C	109.5
O2^i^—Ce1—C9	117.46 (17)	H12B—C12—H12C	109.5
O8—Ce1—C9	93.97 (17)	O8—C13—N2	125.4 (7)
O3—Ce1—C9	25.29 (16)	O8—C13—H13	117.3
O4—Ce1—C9	26.02 (16)	N2—C13—H13	117.3
O5^i^—Ce1—C9	124.18 (15)	N2—C14—H14A	109.5
O1—Ce1—C8^ii^	67.83 (15)	N2—C14—H14B	109.5
O5—Ce1—C8^ii^	102.05 (15)	H14A—C14—H14B	109.5
O7—Ce1—C8^ii^	145.03 (17)	N2—C14—H14C	109.5
O6—Ce1—C8^ii^	23.26 (14)	H14A—C14—H14C	109.5
O2^i^—Ce1—C8^ii^	73.98 (16)	H14B—C14—H14C	109.5
O8—Ce1—C8^ii^	143.82 (16)	N2—C15—H15A	109.5
O3—Ce1—C8^ii^	89.90 (15)	N2—C15—H15B	109.5
O4—Ce1—C8^ii^	102.32 (16)	H15A—C15—H15B	109.5
O5^i^—Ce1—C8^ii^	24.66 (13)	N2—C15—H15C	109.5
C9—Ce1—C8^ii^	99.56 (17)	H15A—C15—H15C	109.5
O1—Ce1—Ce1^i^	60.72 (10)	H15B—C15—H15C	109.5
O5—Ce1—Ce1^i^	42.67 (9)	C10—N1—C11	121.7 (7)
O7—Ce1—Ce1^i^	116.20 (11)	C10—N1—C12	120.9 (7)
O6—Ce1—Ce1^i^	82.87 (9)	C11—N1—C12	117.3 (6)
O2^i^—Ce1—Ce1^i^	70.56 (10)	C13—N2—C15	121.6 (7)
O8—Ce1—Ce1^i^	109.76 (11)	C13—N2—C14	122.2 (7)
O3—Ce1—Ce1^i^	134.19 (10)	C15—N2—C14	116.2 (7)
O4—Ce1—Ce1^i^	157.85 (10)	C1—O1—Ce1	140.8 (4)
O5^i^—Ce1—Ce1^i^	35.43 (8)	C1—O2—Ce1^i^	126.5 (4)
C9—Ce1—Ce1^i^	156.13 (14)	C9—O3—Ce1	93.2 (4)
C8^ii^—Ce1—Ce1^i^	59.61 (12)	C9—O4—Ce1	92.3 (4)
O2—C1—O1	125.5 (6)	C8^vi^—O5—Ce1	164.1 (4)
O2—C1—C2	118.6 (6)	C8^vi^—O5—Ce1^i^	85.9 (3)
O1—C1—C2	115.9 (5)	Ce1—O5—Ce1^i^	101.90 (14)
C7—C2—C3	119.0 (6)	C8^ii^—O6—Ce1	103.0 (4)
C7—C2—C1	122.6 (5)	C10—O7—Ce1	145.6 (4)
C3—C2—C1	118.3 (6)	C13—O8—Ce1	130.4 (5)
C4—C3—C2	120.2 (6)		

Symmetry codes: (i) −*x*+1, −*y*+2, −*z*+1; (ii) −*x*+3/2, *y*+1/2, −*z*+3/2; (iii) −*x*+2, −*y*+2, −*z*+1; (iv) −*x*+3/2, *y*−1/2, −*z*+3/2; (v) *x*+1/2, −*y*+3/2, *z*+1/2; (vi) *x*−1/2, −*y*+3/2, *z*−1/2.
